# Polymerizable Ionic Liquid Crystals Comprising Polyoxometalate Clusters toward Inorganic-Organic Hybrid Solid Electrolytes

**DOI:** 10.3390/polym9070290

**Published:** 2017-07-20

**Authors:** Takeru Ito, Saki Otobe, Tatsuma Oda, Tatsuhiro Kojima, Seiji Ono, Masayuki Watanabe, Yoshiki Kiyota, Toshiyuki Misawa, Shinichi Koguchi, Masashi Higuchi, Masaki Kawano, Yu Nagase

**Affiliations:** 1 Department of Chemistry, School of Science, Tokai University, 4-1-1 Kitakaname, Hiratsuka 259-1292, Japan; sob0406qwer@gmail.com (S.O.); y-kiyota@rigaku.co.jp (Y.K.); t.misawa3303@gmail.com (T.M.); koguchi@tokai-u.jp (S.K.); 2 Department of Applied Chemistry, School of Engineering, Tokai University, Hiratsuka, Kanagawa 259-1292, Japan; tatsuma.oda@gmail.com (T.O.); 6bajm016@mail.u-tokai.ac.jp (S.O.); baseball789643@gmail.com (M.W.); mhig@tokai-u.jp (M.H.); 3 Division of Advanced Materials Science, Pohang University of Science and Technology (POSTECH), Pohang 790-784, Korea; kojimat15@chem.sci.osaka-u.ac.jp (T.K.); mkawano@chem.titech.ac.jp (M.K.); 4 Department of Chemistry, Graduate School of Science, Osaka University, Toyonaka, Osaka 560-0043, Japan; 5 Department of Chemistry, School of Science, Tokyo Institute of Technology, 2-12-1 Ookayama, Meguro-ku, Tokyo 152-8550, Japan

**Keywords:** inorganic-organic hybrid, polymer, ionic liquid, polyoxometalate, conductivity

## Abstract

Solid electrolytes are crucial materials for lithium-ion or fuel-cell battery technology due to their structural stability and easiness for handling. Emergence of high conductivity in solid electrolytes requires precise control of the composition and structure. A promising strategy toward highly-conductive solid electrolytes is employing a thermally-stable inorganic component and a structurally-flexible organic moiety to construct inorganic-organic hybrid materials. Ionic liquids as the organic component will be advantageous for the emergence of high conductivity, and polyoxometalate, such as heteropolyacids, are well-known as inorganic proton conductors. Here, newly-designed ionic liquid imidazolium cations, having a polymerizable methacryl group (denoted as MAImC_1_), were successfully hybridized with heteropolyanions of [PW_12_O_40_]^3−^ (PW_12_) to form inorganic-organic hybrid monomers of MAImC_1_-PW_12_. The synthetic procedure of MAImC_1_-PW_12_ was a simple ion-exchange reaction, being generally applicable to several polyoxometalates, in principle. MAImC_1_-PW_12_ was obtained as single crystals, and its molecular and crystal structures were clearly revealed. Additionally, the hybrid monomer of MAImC_1_-PW_12_ was polymerized by a radical polymerization using AIBN as an initiator. Some of the resulting inorganic-organic hybrid polymers exhibited conductivity of 10^−4^ S·cm^−1^ order under humidified conditions at 313 K.

## 1. Introduction

The development of highly-conductive solid electrolytes is crucial for innovative battery technology, such as lithium-ion and fuel-cell batteries [1,2,3,4], which are now being applied to power sources of motor vehicles working at intermediate temperature of 373 to 573 K. However, they demand highly-conductive solid electrolyte to overcome some drawbacks when used at intermediate temperatures. In most lithium-ion batteries, flammable organic-liquid electrolytes are used, which should be substituted for safer solid electrolytes. Polymer electrolyte membrane fuel cells (PEMFC) use proton-conducting fluorocarbon polymers (Nafion) exhibiting high conductivity only under humidified conditions below 373 K, and highly-conductive solid electrolytes working at intermediate temperatures without humidity are demanded. However, present solid electrolytes, to date, are insufficient in their conductivities. 

A promising option toward highly-conductive solid electrolytes is to employ a thermally-stable inorganic component and a structurally-flexible organic moiety to construct inorganic-organic hybrid conductors [5,6]. Ionic liquids exhibit characteristic conductive properties [7,8,9], and enable the construction of conductive materials [10,11,12,13]. 

As for the inorganic moiety, the vast series of polyoxometalate (POM) inorganic clusters have unique redox properties [14,15,16,17,18,19]. Heteropolyacids, one category of POMs, are well known as highly proton-conductive materials [20,21,22]. However, the application of heteropolyacids is limited due to their hygroscopicity. Hybridization of heteropolyacids with polymers by simple combining has been reported for the emergence of anhydrous proton conductivity at intermediate temperatures [23,24], while precise control of the material’s structure and composition is rather difficult. Hybrid polymers utilizing precisely-synthesized POM anions with a covalently-grafted polymerizable group have been realized [25,26,27,28,29,30,31,32,33,34,35,36], however, this kind of organically-modified POM is limited. Polymerizable organic cations have been rarely employed to construct POM hybrid polymers [37,38], and have not been hybridized with heteropolyacids. 

**Figure 1:**
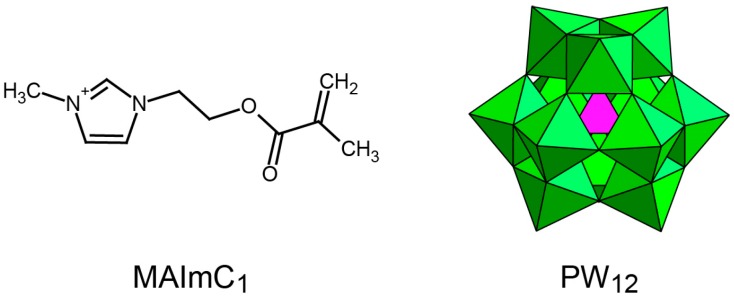
Molecular structure of the MAImC_1_ cation (**left**) and the dodecatungstophosphate (PW_12_) anion (**right**).

Here, conductive inorganic-organic hybrid monomer and polymers were synthesized by using newly-designed ionic liquid imidazolium cations having a polymerizable methacryl group ([{CH_2_=C(CH_3_)COO(CH_2_)_2_}C_3_H_3_N_2_(CH_3_)]^+^, denoted as MAImC_1_, Figure 1). MAImC_1_ was successfully hybridized with heteropolyoxometalate (dodecatungstophosphate, [PW_12_O_40_]^3−^(PW_12_), Figure 1) to obtain a hybrid monomer of MAImC_1_-PW_12_ as single crystals. Radical polymerization of MAImC_1_-PW_12_ hybrid monomer enabled the syntheses of several hybrid homopolymers (P-MAImC_1_-PW_12_) and copolymers (CP-MAImC_1_-PW_12_), some of which exhibited 5.7 × 10^−4^ S·cm^−1^ under humidified conditions at 313 K. 

## 2. Materials and Methods

### 2.1. Materials

All chemical reagents, except for ionic liquid imidazolium cations, were purchased from Wako Pure Chemical Industries, Ltd. (Wako) (Tokyo, Japan) and Tokyo Chemical Industry Co., Ltd. (TCI) (Tokyo, Japan). 2,2′-Azobis(isobutyronitrile) (AIBN, Wako) was purified by recrystallization from ethanol before use, and the other reagents were employed as received. MAImC_1_ and [{CH_2_=C(CH_3_)COO(CH_2_)_2_}C_3_H_3_N_2_(C_8_H_17_)]^+^ (MAImC_8_) were prepared as iodide salt (MAImC_1_-I) and bromide salt (MAImC_8_-Br), respectively, according to the procedures described below. 

### 2.2. Genaral Methods for Characterization

IR spectra (as a KBr pellet) were recorded on a Jasco FT/IR-4200ST spectrometer (JASCO Corporation, Tokyo, Japan). Powder X-ray diffraction (XRD) patterns were measured with a Rigaku MiniFlex300 diffractometer (Rigaku Corporation, Tokyo, Japan) by using Cu Kα radiation (*λ* = 1.54056 Å) at ambient temperature. CHN elemental analyses were performed with a PerkinElmer 2400II elemental analyzer. 

Solution ^1^H NMR spectroscopy was conducted with a Bruker AVANCE-500 NMR spectrometer (Bruker Corporation, Yokohama, Japan) (500 MHz) at room temperature. Gel permeation chromatography (GPC) was carried out to determine the number-average (*M*_n_) and weight-average (*M*_w_) molecular weights with a Tosoh GPC system (Tosoh Corporation, Tokyo, Japan) equipped with four columns of TSK gels, Multipore HXL-M, and a Tosoh RI-8010 detector (Tosoh Corporation, Tokyo, Japan) with a Tosoh CCPD pump (Tosoh Corporation, Tokyo, Japan), using DMF solution containing 20 mM LiBr as an eluent at a flow rate of 1.0 mL min^−1^. GPC was also conducted for THF-soluble polymers with a Tosoh HLC-8320GPC (Tosoh Corporation, Tokyo, Japan) equipped with four columns of TSK gels, and a Super-Multipore HZ-H, using THF as an eluent at a flow rate of 1.0 mL min^−1^. Standard polystyrenes were used to calibrate the molecular weights. Differential scanning calorimetry (DSC) and thermal gravimetric analysis (TGA) were carried out on a Seiko Instruments DSC-6200 (Seiko Instruments Inc., Chiba, Japan) and TG/DTA-6200 (Seiko Instruments Inc., Chiba, Japan), respectively, at a heating rate of 10 K·min^−1^ under a nitrogen atmosphere.

Conductivity measurements were carried out by the alternating current (AC) impedance method. Pelletized powder samples sandwiched with Pt electrodes were employed. Conductivities under ambient atmosphere of relative humidity (RH) ~30% and fully-humidified conditions of RH 95% were measured in a frequency range from 20 to 1.0 × 10^6^ Hz using an Agilent technologies 4284A (Keysight Technologies, California, USA) or a HIOKI 3532-50 inductance-capacitance-resistance (LCR) (HIOKI E.E. Corporation, Nagano, Japan) meter coupled with an Espec SH-641 (ESPEC Corporation, Osaka, Japan) bench-top type temperature and humidity chamber. 

### 2.3. Synthesis

#### 2.3.1. Synthesis of MAImC_1_-I

##### Synthesis of 1-(2-Chloroethyl)-1*H*-imidazole

The solution of imidazole 2.04 g (30 mmol; TCI, purity > 98.0%) in 1,2-dichloroethane (30 mL; TCI, purity > 99.5%) was added to tetrabutylammonium bromide 0.20 g (0.6 mmol) and K_2_CO_3_ 0.82 g (6 mmol). The result mixture was stirred at 358 K for 5 h. The mixture was extracted with CHCl_3_, and the organic layer was washed with water, dried (MgSO_4_) and evaporated. The product was isolated by silica gel column chromatography to give the title compound (1.47 g, 37%) as a colorless liquid. ^1^H NMR (500 MHz, CDCl_3_) δ 3.76 (t, *J* = 10 Hz, 2H), 4.28(t, *J* = 10 Hz, 2H), 6.98 (s, 1H), 7.10 (s, 1H), 7.54 (s, 1H).

##### Synthesis of 2-(1*H*-Imidazol-1-yl) Ethyl Methacrylate

The solution of 1-(2-chloroethyl)-1*H*-imidazole 1.13 g (13.2 mmol) in THF (25 mL) was added to methacrylic acid 1.47 g (11.2 mmol; TCI, purity > 99.0%) and K_2_CO_3_ 3.58 g (25.9 mmol). The resulting mixture was stirred at 339 K overnight. The mixture was extracted with CHCl_3_, and the organic layer was washed with water, dried (MgSO_4_), and evaporated. The product was isolated by silica gel column chromatography to give 1.70 g (84%) of the title compound as a colorless liquid. ^1^H NMR (500 MHz, CDCl_3_) δ 1.93 (s, 3H), 4.25 (t, *J* = 5 Hz, 3H), 4.40 (t, *J* = 5 Hz, 3H), 5.61 (s, 1H), 6.10 (s, 1H), 6.96 (s, 1H), 7.08 (s, 1H), 7.51 (s, 1H).

##### Synthesis of 1-(2-(Methacryloyloxy) Ethyl)-3-Methyl-1*H*-Imidazol-3-ium Iodide (MAImC_1_-I)

The solution of 2-(1*H*-imidazol-1-yl) ethyl methacrylate 10 g (55.6 mmol) in 39.4 g (278 mmol; TCI, purity > 99.5%) iodomethane was stirred at 333 overnight. The product was isolated by silica gel column chromatography to give 8.45 g (52%) of the title compound as a brown liquid. ^1^H NMR (500 MHz, CDCl_3_) δ 1.85 (s, 3H), 3.86 (s, 3H), 4.43 (t, *J* = 5.5 Hz, 3H), 4.52 (t, *J* = 5.5 Hz, 3H), 5.72 (s, 1H), 6.04 (s, 1H), 7.71 (s, 1H), 7.79 (s, 1H), 9.15 (s, 1H).

##### Synthesis of 1-(2-(Methacryloyloxy) Ethyl)-3-Octyl-1*H*-Imidazol-3-ium Bromide (MAImC_8_-Br)

The solution of 2-(1*H*-imidazol-1-yl) ethyl methacrylate 0.61 g (3.4 mmol) in 1-bromooctane 1.15 g (6 mmol; TCI, purity > 98.0%) was stirred at 333 K overnight. The product was isolated by silica gel column chromatography to give 1.00 g (80%) of the title compound as a yellow liquid. ^1^H NMR (500 MHz, DMSO-*d*_6_) δ 0.86 (t, *J* = 6.91 Hz, 3H), 1.25 (m, 10H), 1.77 (m, 2H), 1.85 (t, *J* = 1.14 Hz, 3H). 4.19 (t, *J* = 6.93 Hz, 2H), 4.48 (m, 2H), 4.54 (t, *J* = 4.41 Hz, 2H). 5.72 (quint., *J* = 1.61 Hz, 1H), 6.03 (t, *J* = 1.13 Hz, 1H), 7.85 (m, 2H), 9.29 (s, 1H).

#### 2.3.2. Synthesis of MAImC_1_-PW_12_ Hybrid Monomer

Dodecatungstophosphoric acid *n* hydrate (H_3_[PW_12_O_40_]·*n*H_2_O (H-PW_12_), 1.70 g (5.3 mmol); Wako (Tokyo, Japan), Special Grade) was dissolved in 10 mL of ethanol, and slight amounts of insoluble solid that sometimes remained were filtered off. To the obtained clear colorless solution was added an ethanol solution (5 mL) of MAImC_1_-I (0.50 g, 1.55 mmol) to form a colorless suspension. The suspension was filtered to result in colorless precipitate, which was washed by water and dried under ambient atmosphere to obtain the as-prepared hybrid monomer of MAImC_1_-PW_12_ (1.3 g, yield: 57%). Colorless block crystals of MAImC_1_-PW_12_ were grown from the synthetic filtrate by keeping at 279 K. As-prepared MAImC_1_-PW_12_ hybrid monomer started to shrink and melt over 353 K. Anal.: Calcd for C_30_H_45_N_6_PW_12_O_46_: C: 10.41, H: 1.31, N: 2.43%. Found: C: 13.70, H: 1.60, N: 2.33%. IR (KBr disk): 3161 (w), 2961 (w), 2926 (w), 1723 (w), 1636 (w), 1560 (w), 1455 (w), 1316 (w), 1295 (w), 1164 (w), 1080 (m), 1046 (w). 978 (s), 896 (m), 808 (s), 738 (w), 649 (w), 622 (w), 597 (w), 516 (w) cm^−1^. 

#### 2.3.3. Syntheses of P-MAImC_1_-PW_12_ Hybrid Homopolymers

In a 50 mL eggplant flask, 1.00 g (0.297 mmol) of MAImC_1_-PW_12_ and 24.4 mg (0.149 mmol) of 2,2′-azobis(isobutyronitrile) (AIBN; Wako, recrystallized) were dissolved in 4.3 mL of DMF. After degassing the flask, it was sealed by a three-way cock, and the mixture was stirred at 353 K for 20 h. Then, the reaction mixture was poured into 100 mL of methanol to precipitate the polymer. The obtained polymer was filtered and dried in vacuo to afford 0.852 g of P-MAImC_1_-PW_12_ (Yield: 85.2%). 

#### 2.3.4. Syntheses of CP-MAImC_1_-PW_12_/BMA Hybrid Copolymers

The radical copolymerizations were carried out for the mixtures of MAImC_1_-PW_12_ and butyl methacrylate (BMA; TCI (Tokyo, Japan), purity > 99.0%) to prepare copolymers with different compositions of monomer units. The typical procedure of copolymerization is described below.

In a 50 mL eggplant flask, 0.536 g (0.152 mmol) of MAImC_1_-PW_12_, 0.536 g (3.77 mmol) of BMA, and 12.7 mg (0.0773 mmol) of AIBN were dissolved in 4.5 mL of DMF. After degassing the flask, it was sealed by a three-way cock, and the mixture was stirred at 353 K for 20 h. Then, the reaction mixture was poured into 120 mL of methanol to precipitate the polymer. The obtained polymer was filtered and dried in vacuo to afford 0.659 g of CP-MAImC_1_-PW_12_/BMA (1:1) (Yield: 61.4%). 

#### 2.3.5. Syntheses of CP-MAImC_1_-PW_12_/MAImC_8_Br Hybrid Copolymers

The similar radical copolymerizations were carried out for the mixtures of MAImC_1_-PW_12_ and MAImC_8_Br to prepare copolymers with different compositions. The typical procedure of copolymerization is described below.

In a 50 mL eggplant flask, 0.805 g (0.239 mmol) of MAImC_1_-PW_12_, 0.805 g (2.16 mmol) of MAImC_8_-Br, and 8.0 mg (0.049 mmol) of AIBN were dissolved in 6.8 mL of DMF. After degassing the flask, it was sealed by a three-way cock, and the mixture was stirred at 353 K for 20 h. Then, the reaction mixture was poured into 150 mL of ethyl acetate to precipitate the polymer. The obtained polymer was filtered and dried in vacuo to afford 1.06 g of CP-MAImC_1_-PW_12_/MAImC_8_Br (1:1) (Yield: 65.8%).

### 2.4. X-ray Crystallography

Single crystal X-ray diffraction data for hybrid monomer of MAImC_1_-PW_12_ was recorded with an ADSC Q210 CCD area detector (Area Detector Systems Corporation, Poway, CA, USA) with synchrotron radiation at the 2D beamline at the Pohang Accelerator Laboratory (PAL). The diffraction images were processed by using HKL3000 (HKL Research, Inc., Charlottesville, VA, USA) [39]. Absorption correction was performed with the program PLATON [40]. The structure was solved by the direct methods using SHELXT version 2014/5 [41] and refined by the full-matrix least-squares method on *F*^2^ using SHELXL version 2014/7 [42]. Further details of the crystal structure investigation may be obtained free of charge from the Cambridge Crystallographic Data Centre, 12 Union Road, Cambridge CB2 1EZ, UK; Fax: (+44) 1223 336 033; or E-Mail: deposit@ccdc.cam.ac.uk (CCDC 1555936).

## 3. Results

### 3.1. Synthesis and Structure of MAImC_1_-PW_12_ Hybrid Monomer

**Scheme 1:**
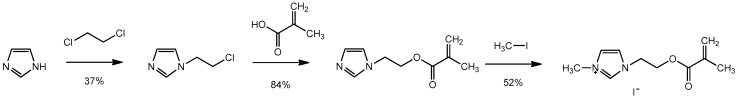
Synthetic route of MAImC_1_-I.

Synthesis of starting polymerizable ionic liquid of MAImC_1_ was obtained as iodide salt according to Scheme 1 (see the Materials and Methods section). 

**Figure 2:**
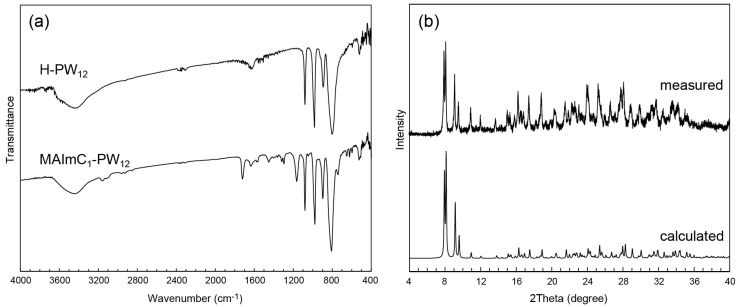
(**a**) IR spectra of H-PW_12_ (upper) and MAImC_1_-PW_12_ hybrid monomers (bottom); and (**b**) powder X-ray diffraction patterns of the MAImC_1_-PW_12_ hybrid monomer measured under ambient atmosphere (upper) and calculated by using the structure obtained by single-crystal X-ray diffraction (bottom).

The colorless precipitate of the MAImC_1_-PW_12_ hybrid monomer was successfully obtained by a cation exchange reaction of dodecatungstophosphoric acid (H-PW_12_) by MAImC_1_, as reported for the syntheses of ionic liquid surfactant-polyoxometalate hybrid crystals [43,44,45]. As shown in Figure 2a, IR spectrum of the MAImC_1_-PW_12_ hybrid monomer exhibited characteristic peaks of dodecatungstophosphate (PW_12_) anion in the range of 400–1100 cm^−1^ [17,46], as well as peaks derived from MAImC_1_ (methylene groups in 2800–3000 cm^−1^ and methacryl group in 1200–1800 cm^−1^). This indicates the successful hybridization of polymerizable MAImC_1_ cations and PW_12_ anions. The XRD pattern of the MAImC_1_-PW_12_ hybrid monomer obtained as the initial precipitate was almost the same as the pattern calculated from the results of the single crystal X-ray analysis (Figure 2b), indicating that the as-prepared precipitate and single crystals of MAImC_1_-PW_12_ had the same composition and structure. Differences in the peak intensity and position of the patterns may be due to the difference in the measurement temperature (powder: ambient temperature, single crystal: 100 K). 

**Figure 3:**
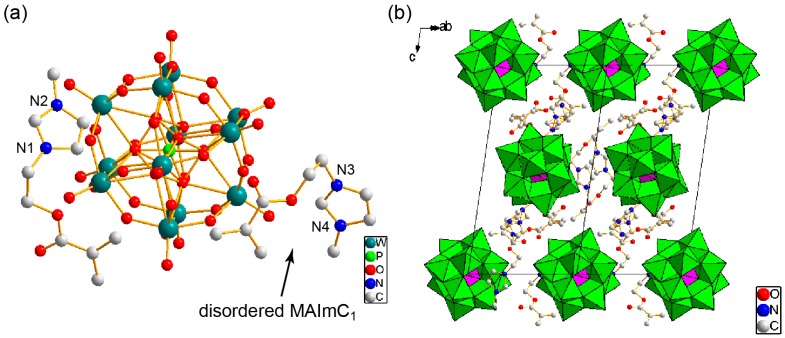
Crystal structure of MAImC_1_-PW_12_ hybrid monomer. H atoms are omitted for clarity: (**a**) asymmetric unit together with PW_12_ anion completed by symmetry operation (0.5 − *x*, 0.5 − *y*, 1 − *z*); and (**b**) packing diagram along the [−1, 1, 0] direction.

The molecular structure was clearly revealed by single-crystal X-ray structure analysis (Table 1, Figure 3). The chemical formula was [{CH_2_=C(CH_3_)COO(CH_2_)_2_}C_3_H_3_N_2_(CH_3_)]_3_[PW_12_O_40_] (MAImC_1_-PW_12_), and three protons of H-PW_12_ were completely exchanged by three MAImC_1_ cations. As shown in Figure 3a, there were two crystallographically-independent MAImC_1_ cations, one of which was disordered near the two-fold axis with a site occupancy of 0.5. The PW_12_ anion lay on the inversion center, and exhibited a common type of disorder for the Keggin anion noted previously [47,48]. 

### 3.2. Synthesis of MAImC_1_-PW_12_ Hybrid Homo- and Copolymers

**Scheme 2:**
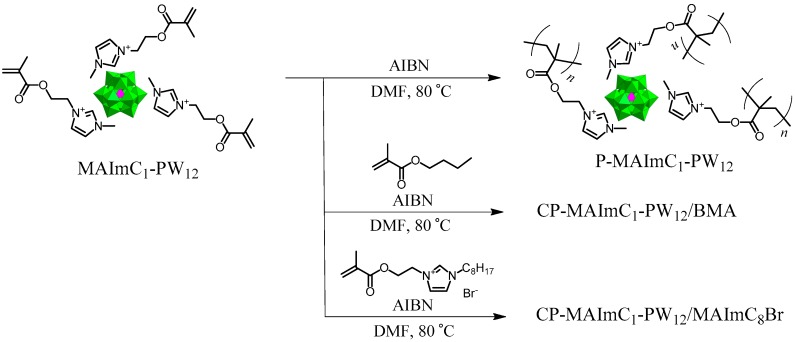
Synthesis of MAImC_1_-PW_12_ hybrid homo- and copolymers.

The radical polymerization of the obtained MAImC_1_-PW_12_ hybrid monomer and the copolymerizations with vinyl monomers, butyl methacrylate (BMA), and MAImC_8_-Br were carried out by using AIBN as an initiator as shown in Scheme 2. The yields and the molecular weights of the obtained polymers are shown in Table 2. MAImC_1_-PW_12_ hybrid monomer was successfully polymerized by using about a half equivalent of AIBN against the monomer, where the homopolymer was soluble in some organic solvents and the weight-average molecular weight was 7960. The homopolymer, P-MAImC_1_-PW_12_, was soluble in DMF, DMSO, and NMP, but insoluble in methanol, chloroform, and THF. On the other hand, the copolymerization of the MAImC_1_-PW_12_ hybrid monomer with BMA and MAImC_8_-Br were conducted using a 1/50 equivalent amount of AIBN against the monomers to yield high molecular weight copolymers. The copolymers of MAImC_1_-PW_12_ with BMA, CP-MAImC_1_-PW_12_/BMA, were widely soluble in chloroform, THF, DMF, and NMP, however, the copolymers of MAImC_1_-PW_12_ with MAImC_8_-Br, CP-MAImC_1_-PW_12_/MAImC_8_Br, were only soluble in NMP. Therefore, GPC measurement of CP-MAImC_1_-PW_12_/MAImC_8_Br could not be conducted. 

**Figure 4:**
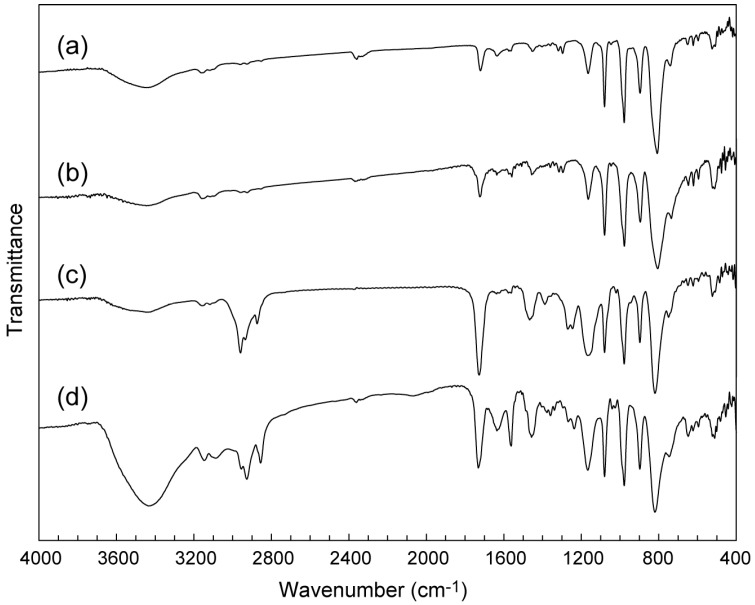
IR spectra of MAImC_1_-PW_12_ hybrids: (**a**) MAImC_1_-PW_12_ hybrid monomer; (**b**) P-MAImC_1_-PW_12_ hybrid homopolymer by using DMF; (**c**) CP-MAImC_1_-PW_12_/BMA hybrid copolymer; and (**d**) CP-MAImC_1_-PW_12_/MAImC_8_Br hybrid copolymer.

IR spectroscopy confirmed successful polymerization of the MAImC_1_-PW_12_ hybrid monomer. IR spectra of MAImC_1_-PW_12_ hybrid homopolymer (Figure 4b) and copolymers (Figure 4c,d) exhibited characteristic peaks of the PW_12_ anion in the range of 400–1100 cm^−1^ as observed for that of the MAImC_1_-PW_12_ hybrid monomer (Figure 4a). 

**Figure 5:**
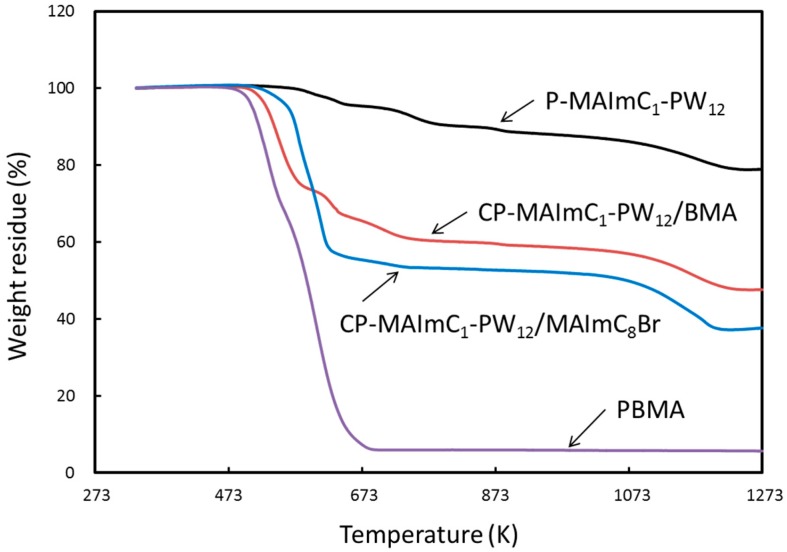
Thermogravimetric curves of MAImC_1_-PW_12_ hybrid homo- and copolymers as compared with poly(butyl methacrylate) (PBMA).

The thermal stability of these hybrid polymers was evaluated by thermogravimetric analysis (TGA), and compared with poly(butyl methacrylate) (PBMA) as shown in Figure 5. It was found from the TGA curves that the thermal degradation of the hybrid homo- and copolymers of MAImC_1_-PW_12_ mainly occurred at around 503–553 K, whereas that of PBMA occurred at just over 473 K. Probably, the thermal degradation of the hybrid polymers would be induced by a decomposition of the organic components, imidazolium groups, at ca. 500 K, whereas the starting temperatures of degradation for all the hybrid polymers were higher than that of purely-organic PBMA polymer. Notably, CP-MAImC_1_-PW_12_/MAImC_8_Br hybrid copolymer exhibiting moderate conductivity (see below) was much more stable than PBMA. It was considered that the weight loss of these hybrid polymers would be derived from the degradation of the polymer side chain. These results indicated that the hybridization with the PW_12_ inorganic anions enhanced the thermal stability of the organic polymers.

**Figure 6:**
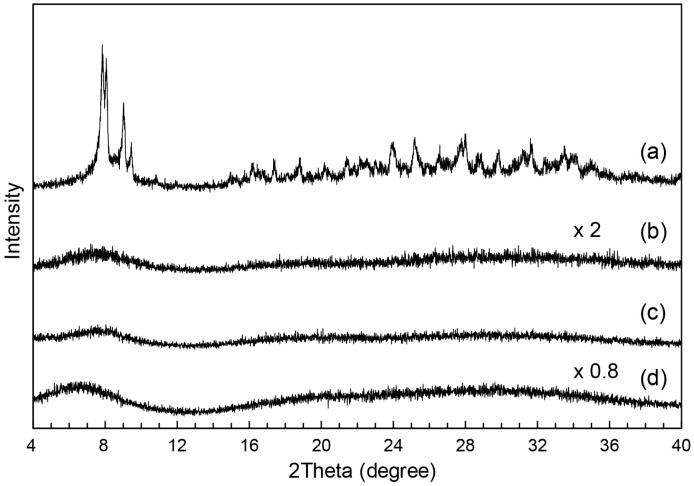
Powder X-ray diffraction patterns of MAImC_1_-PW_12_ hybrids: (**a**) P-MAImC_1_-PW_12_ hybrid homopolymer prepared by using DMSO; (**b**) P-MAImC_1_-PW_12_ hybrid homopolymer prepared by using DMF; (**c**) CP-MAImC_1_-PW_12_/BMA hybrid copolymer; and (**d**) CP-MAImC_1_-PW_12_/MAImC_8_Br hybrid copolymer.

Figure 6 shows powder XRD patterns of the MAImC_1_-PW_12_ hybrid homo- and copolymers. As shown in Figure 6a, MAImC_1_-PW_12_ hybrid homopolymer prepared with DMSO exhibited an XRD pattern similar to that of crystalline MAImC_1_-PW_12_ hybrid monomer (Figure 2b). This indicates that the polymerization did not proceed sufficiently, being consistent with the molecular weight estimated by GPC (Table 2). On the contrary, the XRD pattern of the MAImC_1_-PW_12_ hybrid homopolymer prepared with DMF (Figure 6b) did not show any peak except for halo patterns typical for amorphous polymer phase, suggesting that the MAImC_1_-PW_12_ hybrid monomer was successfully polymerized. Both XRD patterns of MAImC_1_-PW_12_ hybrid copolymers (Figure 6c,d) showed halo peaks typical for an amorphous polymer phase, supported by the GPC results (Table 2). 

### 3.3. Conductivity of MAImC_1_-PW_12_ Hybrid Monomer and Polymers

**Figure 7:**
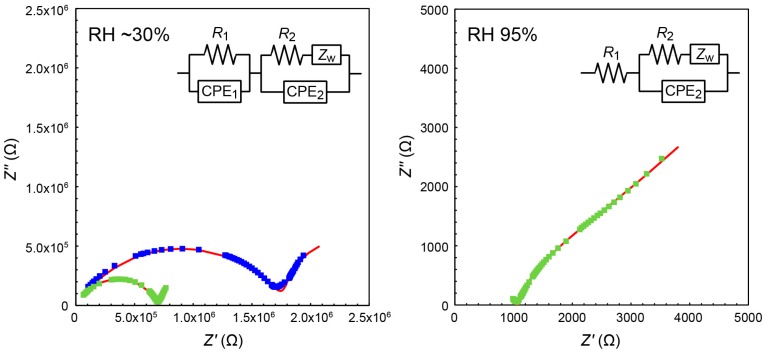
Nyquist plots of CP-MAImC_1_-PW_12_/MAImC_8_Br (1:1) hybrid copolymer at 298 K (blue) and 313 K (green) without additional humidity (RH ~30%) (**left**) and with additional humidity of RH 95% (**right**). The simulated spectra (red line) are based on an equivalent electronic circuit in the figure.

Table 3 shows conductivity values of MAImC_1_-PW_12_ hybrid monomer and polymers measured under less- or fully-humidified conditions. Most samples, unfortunately, exhibited rather low conductivity below the order of 10^−7^ S·cm^−1^ despite the degree of polymerization and/or presence of humidity. However, the copolymer of MAImC_1_-PW_12_ with MAImC_8_-Br (CP-MAImC_1_-PW_12_/MAImC_8_Br (1:1)) exhibited much better conductivity. The Nyquist plots (Figure 7) changed drastically with or without additional humidification, and the conductivities were estimated by the resistance (*R*_1_) simulated by the equivalent circuits depicted in Figure 7. Table 4 shows estimated parameters with these equivalent circuits. The conductivity under relative humidity (denoted as RH) of 95% was 5.7 × 10^−4^ S·cm^−1^, which was a moderate value at the lower temperature (313 K). The conductivity of the CP-MAImC_1_-PW_12_/MAImC_8_Br hybrid copolymer depended on the ratio of MAImC_1_-PW_12_ hybrid monomer and MAImC_8_-Br comonomer. The higher the ratio of MAImC_1_-PW_12_ hybrid monomer to MAImC_8_-Br comonomer, the lower the conductivity was: the conductivity of CP-MAImC_1_-PW_12_/MAImC_8_Br (3:1) with additional humidity (3.5 × 10^−6^ S·cm^−1^) was two orders of magnitude lower than that of CP-MAImC_1_-PW_12_/MAImC_8_Br (1:1) (Table 3). Therefore, the moderate conductivity of CP-MAImC_1_-PW_12_/MAImC_8_Br under humidified conditions was derived from polymerized MAImC_8_-Br moiety, which was confirmed by high conductivity (3.2 × 10^−3^ S·cm^−1^) of homopolymer of MAImC_8_-Br (P-MAImC_8_Br) as shown in Table 3. However, P-MAImC_8_Br irreversibly expanded under humidified conditions to result in a gel-like material, and the durability of P-MAImC_8_Br against humidity was quite low, and the conductivity measurement was able only one time. On the other hand, CP-MAImC_1_-PW_12_/MAImC_8_Br hybrid copolymers endured the presence of humidity, and the conductivity measurements were carried out repeatedly. Therefore, the hybridization of MAImC_1_-PW_12_ with MAImC_8_Br seems crucial for the increase in durability of P-MAImC_8_Br homopolymer and the emergence of moderate conductivity. 

## 4. Discussion

The newly-designed polymerizable ionic liquid cation of MAImC_1_ was successfully hybridized with heteropolyanion of dodecatungstophosphate (PW_12_). The inorganic-organic hybrid momomer of MAImC_1_-PW_12_ was revealed to be polymerized by a radical polymerization using AIBN as an initiator. Both hybrid monomer and polymers contained imidazolium moieties, and were expected to work as ionic or proton conductors [10,11,12,13,23]. 

These MAImC_1_-PW_12_ hybrid monomer and polymers have an advantage to the generality on the selection of polyoxometalate anions and ionic liquid cations. In our compounds, the polymerizable ionic liquid moiety is a cationic species and interacts with heteropolyanions to form ionic hybrid monomer compounds [37,38]. In principle, the organic ionic liquid moiety can be flexibly designed in terms of organic syntheses, and the inorganic polyoxometalate anions can be variously selected, indicating more versatility than other polyoxometalate-polymer systems using polyoxometalate with the organic moiety grafted by covalent bonding [25,26,27,28,29,30,31,32,33,34,35,36]. Additionally, the MAImC_1_-PW_12_ hybrid monomer can be obtained as single crystals [28,29,30,31,32], which enabled unambiguous characterization of the key starting material. Other combinations of the polymerizable ionic liquid moiety and polyoxometalate are now investigated. 

The carrier for the conduction of CP-MAImC_1_-PW_12_/MAImC_8_Br is unclear. The conductivity increased under the presence of water vapor, indicating the water molecules may assist the moving of carriers. The proton conductivity often increases under the presence of water vapor [3]. Therefore, the carrier in CP-MAImC_1_-PW_12_/MAImC_8_Br is suggested to be proton. The proton may be derived from water molecules, since the MAImC_1_-PW_12_ hybrid monomer and polymers have no residual protons, as revealed by single-crystal structure analysis. As mentioned above, the moderate conductivity of CP-MAImC_1_-PW_12_/MAImC_8_Br (1:1) (5.7 × 10^−4^ S·cm^−1^ at 313 K) was derived from the MAImC_8_-Br moiety (Table 3). The water molecules may loosen the polymer chain of the polymerized MAImC_8_-Br moiety, and the carrier species could move more easily under the presence of humidity. The CP-MAImC_1_-PW_12_/MAImC_8_Br hybrid copolymer had good durability against water vapor, while the purely organic P-MAImC_8_Br polymer was quite sensitive against humidity. The increase in the durability against humidity will be derived from the introduction of PW_12_ anions into P-MAImC_8_Br, and the hybridization with the inorganic moiety has been revealed to be effective for possible inorganic-organic hybrid solid electrolyte [49]. 

## 5. Conclusions

Polymerizable ionic liquid (MAImC_1_) was utilized to construct the inorganic-organic hybrid monomer with heteropolyanions (PW_12_). The hybrid monomer was synthesized by a cation exchange reaction, and obtained as single crystals. This new hybrid monomer was successfully polymerized by a radical polymerization to form several types of homo- and copolymers. Copolymerization of MAImC_1_-PW_12_ with MAImC_8_-Br resulted in the formation of a possible solid electrolyte. The conductivity of the inorganic-organic hybrid copolymer had a moderate value of 5.7 × 10^−4^ S·cm^−1^ at 313 K under humidified conditions. 

## Figures and Tables

**Table 1:** Crystallographic data of the MAImC_1_-PW_12_ hybrid monomer.

Compound	MAImC_1_-PW_12_
Chemical formula	C_30_H_45_N_6_PW_12_O_46_
Formula weight	3462.87
Crystal system	Monoclinic
Space group	*C*2/*c* (No. 15)
*a* (Å)	18.9467(2)
*b* (Å)	14.6994(2)
*c* (Å)	22.9023(4)
α (°)	90.0000
β (°)	103.390(1)
γ (°)	90.0000
*V* (Å^3^)	6205.02(15)
*Z*	4
ρ_calcd_ (g cm^−3^)	3.707
*T* (K)	100(2)
Wavelength (Å)	0.70000
μ (mm^−1^)	20.615
No. of reflections measured	29,677
No. of independent reflections	8833
*R*_int_	0.0525
No. of parameters	505
*R*_1_ (*I* > 2σ(*I*))	0.0641
*wR*_2_ (all data)	0.1651

**Table 2:** Results of polymerizations.

Code	Yield (%)	*M*_n_^1^ × 10^−3^	*M*w ^1^ × 10^−3^	*M*_w_/*M*_n_
P-MAImC_1_-PW_12_	85	7.47 ^(a)^	7.96 ^(a)^	1.07 ^(a)^
CP-MAImC_1_-PW_12_/BMA (1:1)	67	35.4 ^(a)^	45.4 ^(a)^	1.28 ^(a)^
CP-MAImC_1_-PW_12_/BMA (1:3)	68	9.88 ^(b)^	12.1 ^(b)^	1.17 ^(b)^
PBMA homopolymer	71	20.1 ^(b)^	28.8 ^(b)^	1.44 ^(b)^

^1^ Number- and weight-average molecular weights (*M*_n_ and *M*_w_) were determined by GPC using ^(a)^ 20 mM LiBr solution in DMF or ^(b)^ THF as an eluent.

**Table 3:** Conductivity values of MAImC_1_-PW_12_ hybrids with or without additional humidity.

Code	Without Additional Humidity (Measured at 298 K) (S·cm^−1^)	Without Additional Humidity (Measured Temperature) (S·cm^−1^)	With Additional Humidity of 95% RH (Measured Temperature) (S·cm^−1^)
MAImC_1_-PW_12_	1.2 × 10^−8^	3.0 × 10^−8^ (327 K)	3.0 × 10^−7^ (327 K)
P-MAImC_1_-PW_12_	8.4 × 10^−10^	2.0 × 10^−7^ (373 K)	7.4 × 10^−6^ (373 K)
CP-MAImC_1_-PW_12_/BMA (1:3)	8.6 × 10^−10^	5.2 × 10^−8^ (373 K)	1.5 × 10^−9^ (373 K)
CP-MAImC_1_-PW_12_/MAImC_8_Br (1:1)	3.3 × 10^−7^	8.4 × 10^−7^ (313 K)	5.7 × 10^−4^ (313 K)
CP-MAImC_1_-PW_12_/MAImC_8_Br (3:1)	6.0 × 10^−8^	7.2 × 10^−8^ (313 K)	3.5 × 10^−6^ (313 K)
P-MAImC_8_Br	1.8 × 10^−6^	9.2 × 10^−6^ (313 K)	3.2 × 10^−3^ (313 K) ^1^

^1^ The durability of the sample against humidity was very low.

**Table 4:** Estimated parameters with the equivalent circuit for CP-MAImC_1_-PW_12_/MAImC_8_Br (1:1).

Circuit Parameter	Without Additional Humidity at 298 K	Without Additional Humidity at 313 K	With Additional Humidity of 95% RH at 313 K
*R*_1_ (Ω)	1.78 × 10^6^	7.06 × 10^5^	1.09 × 10^3^
CPE_1_-*P* ^1^	2.70 × 10^−10^	1.23 × 10^−10^	-
CPE_1_-*n* ^1^	0.627	0.714	-
*R*_2_ (Ω)	2.77 × 10^7^	4.98 × 10^9^	2.98 × 10^3^
CPE_2_-*P* ^1^	4.79 × 10^−8^	1.51 × 10^−7^	3.26 × 10^−6^
CPE_2_-*n* ^1^	0.757	0.759	0.76
σ ^2^	2.90 × 10^4^	2.90 × 10^4^	2.90 × 10^4^

^1^ Constant phase element (CPE) defined as *Z*(*ω*) = *P*^−1^(*j*·*ω*)^−*n*^. *P* in Ω^−1^ s^−*n*^, *n* in non-dimensional. ^2^ Warburg impedance defined as *Z*_w_ = (1 − *j*)σ(*ω*)^−1/2^. σ in Ω s^−1/2^.

## Supplementary Materials

Supplementary materials are available online at www.mdpi.com/2073-4360/9/7/290/s1.

## References

[1] Whittingham M.S. (2004). Lithium batteries and cathode materials. Chem. Rev..

[2] Goodenough J.B., Park K.-S. (2013). The Li-ion rechargeable battery: A perspective. J. Am. Chem. Soc..

[3] Steele B.C.H., Heinzel A. (2001). Materials for fuel-cell technologies. Nature.

[4] Yoon M., Suh K., Natarajan S., Kim K. (2013). Proton conduction in metal-organic frameworks and related modularly built porous solids. Angew. Chem. Int. Ed..

[5] Coronado E., Gómez-García C.J. (1998). Polyoxometalate-based molecular materials. Chem. Rev..

[6] Coronado E., Giménez-Saiz C., Gómez-García C.J. (2005). Recent advances in polyoxometalate-containing molecular conductors. Coord. Chem. Rev..

[7] Welton T. (1999). Room-temperature ionic liquids. Solvents for synthesis and catalysis. Chem. Rev..

[8] Wasserscheid P., Keim W. (2000). Ionic liquids-new “solutions” for transition maetal catalysis. Angew. Chem. Int. Ed..

[9] Haumann M., Riisager A. (2008). Hydroformylation in room temperature ionic liquids (RTILs): Catalyst and process developments. Chem. Rev..

[10] Kato T., Mizoshita N., Kishimoto K. (2006). Functional liquid-crystalline assemblies: Self-organized soft materials. Angew. Chem. Int. Ed..

[11] Nishimura N., Ohno H. (2014). 15th anniversary of polymerised ionic liquids. Polymer.

[12] Bureekaew S., Horike S., Higuchi M., Mizuno M., Kawamura T., Tanaka D., Yanai N., Kitagawa S. (2009). One-dimensional imidazole aggregate in aluminium porous coordination polymers with high proton conductivity. Nat. Mater..

[13] Watanabe M., Thomas M.L., Zhang S., Ueno K., Yasuda T., Dokko K. (2017). Application of ionic liquids to energy storage and conversion materials and devices. Chem. Rev..

[14] Long D.-L., Burkholder E., Cronin L. (2007). Polyoxometalate clusters, nanostructures and materials: From self assembly to designer materials and devices. Chem. Soc. Rev..

[15] Nyman M. (2011). Polyoxoniobate chemistry in the 21st century. Dalton Trans..

[16] Proust A., Matt B., Villanneau R., Guillemot G., Gouzerh P., Izzet G. (2012). Functionalization and post-functionalization: A step towards polyoxometalate-based materials. Chem. Soc. Rev..

[17] Okuhara T., Mizuno N., Misono M. (1996). Catalytic chemistry of heteropoly compounds. Adv. Catal..

[18] Yamase T. (1998). Photo- and electrochromism of polyoxometalates and related materials. Chem. Rev..

[19] Sadakane M., Steckhan E. (1998). Electrochemical properties of polyoxometalates as electrocatalysts. Chem. Rev..

[20] Nakamura O., Kodama T., Ogino I., Miyake Y. (1979). High-conductivity solid proton conductors: Dodecamolybdophosphoric acid and dodecatungstophosphoric acid crystals. Chem. Lett..

[21] Bourlinos A.B., Raman K., Herrera R., Zhang Q., Archer L.A., Giannelis E.P. (2004). A liquid derivative of 12-tungstophosphoric acid with unusually high conductivity. J. Am. Chem. Soc..

[22] Wu X., Tong X., Wu Q., Ding H., Yan W. (2014). Reversible phase transformation-type electrolyte based on layered shape polyoxometalate. J. Mater. Chem. A.

[23] Honma I., Yamada M. (2007). Bio-inspired membranes for advanced polymer electrolyte fuel cells. Anhydrous proton-conducting membrane via molecular self-assembly. Bull. Chem. Soc. Jpn..

[24] Oh S.-Y., Yoshida T., Kawamura G., Muto H., Sakai M., Matsuda A. (2010). Inorganic-organic composite electrolytes consisting of polybenzimidazole and Cs-substituted heteropoly acids and their application for medium temperature fuel cells. J. Mater. Chem..

[25] Judeinstein P. (1992). Synthesis and properties of polyoxometalates based inorganic-organic polymers. Chem. Mater..

[26] Mayer C.R., Thouvenot R., Lalot T. (2000). New hybrid covalent networks based on polyoxometalates: Part 1. Hybrid networks based on poly(ethyl methacrylate) chains covalently cross-linked by heteropolyanions: Synthesis and swelling properties. Chem. Mater..

[27] Mayer C.R., Thouvenot R., Lalot T. (2000). Hybrid hydrogels obtained by the copolymerization of acrylamide with aggregates of methacryloyl derivatives of polyoxotungstates. A comparison with polyacrylamide hydrogels with trapped aggregates. Macromolecules.

[28] Moore A.R., Kwen H., Beatty A.M., Eric A., Maatta E.A. (2000). Organoimido-polyoxometalates as polymer pendants. Chem. Commun..

[29] Lu M., Xie B., Kang J., Chen F.-C., Yang Y., Peng Z. (2005). Synthesis of main-chain polyoxometalate-containing hybrid polymers and their applications in photovoltaic cells. Chem. Mater..

[30] Xu B., Lu M., Kang J., Wang D., Brown J., Peng Z. (2005). Synthesis and optical properties of conjugated polymers containing polyoxometalate clusters as side-chain pendants. Chem. Mater..

[31] Hasegawa T., Shimizu K., Seki H., Murakami H., Yoshida S., Yoza K., Nomiya K. (2007). Polymerizable inorganic-organic hybrid: Syntheses and structures of mono-lacunary Dawson polyoxometalate-based olefin-containing organosilyl derivatives. Inorg. Chem. Commun..

[32] Hasegawa T., Murakami H., Shimizu K., Kasahara Y., Yoshida S., Kurashina T., Seki H., Nomiya K. (2008). Formation of inorganic protonic-acid polymer via inorganic-organic hybridization: Synthesis and characterization of polymerizable olefinic organosilyl derivatives of mono-lacunary Dawson polyoxometalate. Inorg. Chim. Acta.

[33] Horan J.L., Genupur A., Ren H., Sikora B.J., Kuo M.-C., Meng F., Dec S.F., Haugen G.M., Yandrasits M.A., Hamrock S.J. (2009). Copolymerization of divinylsilyl-11-silicotungstic acid with butyl acrylate and hexanediol diacrylate: Synthesis of a highly proton-conductive membrane for fuel-cell applications. ChemSusChem.

[34] Han Y., Xiao Y., Zhang Z., Liu B., Zheng P., He S., Wang W. (2009). Synthesis of polyoxometalate-polymer hybrid polymers and their hybrid vesicular assembly. Macromolecules.

[35] Miao W.-K., Yan Y.-K., Wang X.-L., Xiao Y., Ren L.-J., Zheng P., Wang C.-H., Ren L.-X., Wang W. (2014). Incorporation of polyoxometalates into polymers to create linear poly(polyoxometalate)s with catalytic function. ACS Macro Lett..

[36] Macdonell A., Johnson N.A.B., Surman A.J., Cronin L. (2015). Configurable nanosized metal oxide oligomers via precise “click” coupling control of hybrid polyoxometalates. J. Am. Chem. Soc..

[37] Li H., Qi W., Li W., Sun H., Bu W., Wu L. (2005). A highly transparent and luminescent hybrid based on the copolymerization of surfactant-encapsulated polyoxometalate and methyl methacrylate. Adv. Mater..

[38] Qi W., Wu L. (2009). Polyoxometalate/polymer hybrid materials: Fabrication and properties. Polym. Int..

[39] Otwinowski Z., Minor W. (1997). Processing of X-ray diffraction data collected in oscillation mode. Methods Enzymol..

[40] Spek A.L. (2009). Structure validation in chemical crystallography. Acta Crystallogr. Sect. D.

[41] Sheldrick G.M. (2015). SHELXT—Integrated space-group and crystal structure determination. Acta Crystallogr. Sect. A.

[42] Sheldrick G.M. (2008). A short history of SHELX. Acta Crystallogr. Sect. A.

[43] Ito T., Ide R., Kosaka K., Hasegawa S., Mikurube K., Taira M., Naruke H., Koguchi S. (2013). Polyoxomolybdate-surfactant layered crystals derived from long-tailed alkylamine and ionic-liquid. Chem. Lett..

[44] Ito T., Mikurube K., Hasegawa K., Matsumoto T., Kosaka K., Naruke H., Koguchi S. (2014). Structural variation in polyoxomolybdate hybrid crystals comprising ionic-liquid surfactants. Crystals.

[45] Kobayashi J., Kawahara R., Uchida S., Koguchi S., Ito T. (2016). Conductive hybrid crystal composed from polyoxomolybdate and deprotonatable ionic-liquid surfactant. Int. J. Mol. Sci..

[46] Rocchiccioli-Deltcheff C., Fournier M., Franck R., Thouvenot R. (1983). Vibrational investigations of polyoxometalates. 2. Evidence for anion-anion interactions in molybdenum(v1) and tungsten(v1) compounds related to the Keggin structure. Inorg. Chem..

[47] Evans H.T., Pope M.T. (1984). Reinterpretation of five recent crystal structures of heteropoly and isopoly complexes: Divanadodecamolybdopbosphate, trivanadoenneamolybdopbospbate, “γ-dodecatungstophospbate”, the dodecamolybdate-dodecamolybdomolybdate blue complex, and dihydrogen decavanadate. Inorg. Chem..

[48] Dong J., Hu J., Chi Y., Lin Z., Zou B., Yang S., Hill C.L., Hu C. (2017). A polyoxoniobate–polyoxovanadate double-anion catalyst for simultaneous oxidative and hydrolytic decontamination of chemical warfare agent simulants. Angew. Chem. Int. Ed..

[49] Herrmann S., Ritchie C., Streb C. (2015). Polyoxometalate—Conductive polymer composites for energy conversion, energy storage and nanostructured sensors. Dalton Trans..

